# Improving Quantum Well Tube Homogeneity Using Strained Nanowire Heterostructures

**DOI:** 10.1021/acsami.2c22591

**Published:** 2023-02-13

**Authors:** Nikesh Patel, H. Aruni Fonseka, Yunyan Zhang, Stephen Church, Ruqaiya Al-Abri, Ana Sanchez, Huiyun Liu, Patrick Parkinson

**Affiliations:** † Department of Physics & Astronomy, Photon Science Institute, The University of Manchester, Oxford Road, Manchester, M13 9PL, United Kingdom; ‡ Department of Physics, University of Warwick, Coventry, CV4 7AL, United Kingdom; § Department of Electronic and Electrical Engineering, University College London, London, WC1E 6BT, United Kingdom; ∥ School of Micro-Nano Electronics, Zhejiang University, Hangzhou, Zhejiang 311200, China

**Keywords:** semiconductor, nanowire, quantum well, homogeneity, heterostructure, high-throughput study

## Abstract

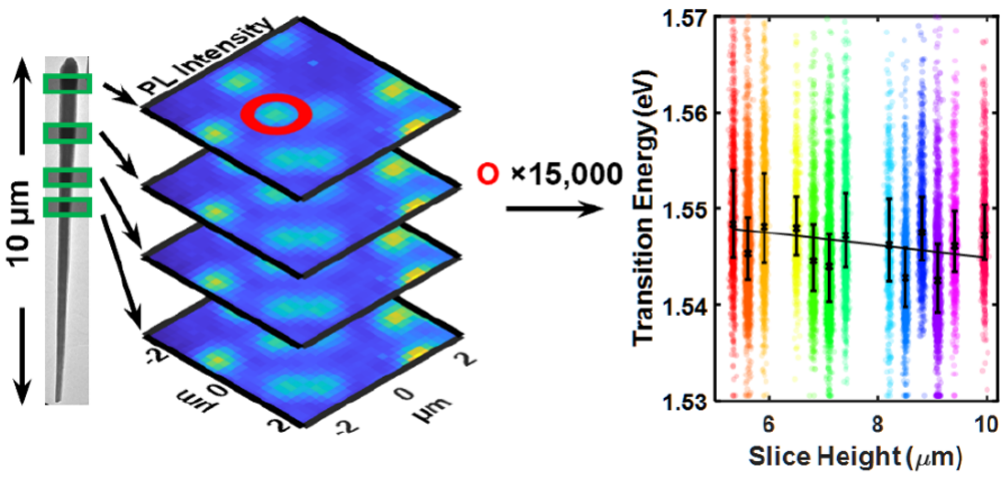

Bottom-up grown nanostructures often suffer from significant dimensional inhomogeneity, and for quantum confined heterostructures, this can lead to a corresponding large variation in electronic properties. A high-throughput characterization methodology is applied to >15,000 nanoskived sections of highly strained GaAsP/GaAs radial core/shell quantum well heterostructures revealing high emission uniformity. While scanning electron microscopy shows a wide nanowire diameter spread of 540_–60_^+60^ nm, photoluminescence reveals a tightly bounded band-to-band transition energy of 1546_–3_^+4^ meV. A highly strained core/shell nanowire design is shown to reduce the dependence of emission on the quantum well width variation significantly more than in the unstrained case.

## Introduction

There is a demand for faster, denser, and increasingly efficient optoelectronic devices for use in next generation optoelectronic devices such as telecommunications^1,2^ and energy harvesting applications.^3^ While planar heterostructure growth has been optimized for decades,^4^ heterostructured bottom-up grown materials such as nanowires^5^ (NWs) are less well controlled due to the complex interplay between local growth environment, a narrow growth window, and a stochastic seeding process.^6^ Optoelectronically homogeneous and defect-free NWs are desired for photonic integrated circuits^7−9^ where material quality has been shown to impact electron mobility^10^ and in photovoltaic cells^3,11−14^ where improved homogeneity directly correlates to total device efficiency and functional yield.^15^ Radial quantum well (QW) heterostructures are one particular candidate for these applications due to ease of tunability and a demonstrated compatibility with highly strained systems.^12^ III–V semiconducting materials are well suited to hosting such heterostructures, as well as possessing excellent waveguiding properties, high carrier mobility, and high optical efficiency.^16,17^ GaAs/GaAsP QW structures are of recent interest, as they avoid or minimize use of aluminum incorporated material such as AlGaAs in the active region, which readily oxidizes, reducing device longevity. In QW systems, intramaterial strain improves carrier confinement^18^ and radiative efficiency^19^ at room temperature, which is particularly important for nanolasers at telecommunication wavelengths.^1^

The relative impact of material inhomogeneity and disorder increases significantly toward nanometer fabrication scales;^20,21^ when considering heterostructures, such as QWs, even monolayer thickness variations can lead to significant shifts in their emission range.^22^ As such, tight control of local growth conditions is considered essential^23−26^ which is particularly difficult for bottom-up grown materials at the wafer-scale. In contrast to control of median emission energy, the systematic optimization of interwire homogeneity requires a statistical interpretation of material properties (such as diameter, emission intensity and wavelength, and local structure separation) that are in turn linked to growth processes. This can be achieved using correlated measurements.^27^ Obtaining a statistically significant data set (of around 10^3^–10^6^ data points per growth) can be cumbersome with manual sequential techniques. Automated high-throughput techniques are required to enable rapid, single-point measurements of individual structures and have been shown to be advantageous for characterizing interwire homogeneity.^28,29^ However, *intrawire* variation in heterostructure properties remains hard to access beyond the single wire level^30^ and no clear methodology has been established that determines both inter- and intrawire homogeneity in quantum systems at a statistically significant scale linking optoelectronic properties to geometry and to optimized growth strategy.

In this work, confocal microphotoluminescence spectroscopy (μPL) and scanning electron microscopy (SEM) are used to compare both intra- and interwire morphological and optoelectronic characteristics of nanoskived^24^ (cross-sectioned) radial GaAsP/GaAs core/shell QW structures. SEM of embedded and sectioned as-grown nanowire ensembles was used to obtain structure diameters and interstructure separation as a function of their location within each wire, which was correlated with electronic properties probed by μPL to investigate the interplay between optoelectronic and morphological character along the NW length. Despite an ∼40% increase in the median diameter along the NW length, only a <1% variation in emission energy is observed. This is attributed to highly homogeneous recombination in the active QW regions arising from both a reduced dependency of emission on well width for a given emission energy and high quality control of growth, demonstrating the benefit of the NW heterostructure platform for industrial-scale applications and the importance of focusing on the highly strained, radially grown QW configuration.

## Methods and Materials

### MBE-Grown Nanowires

**Figure 1:**
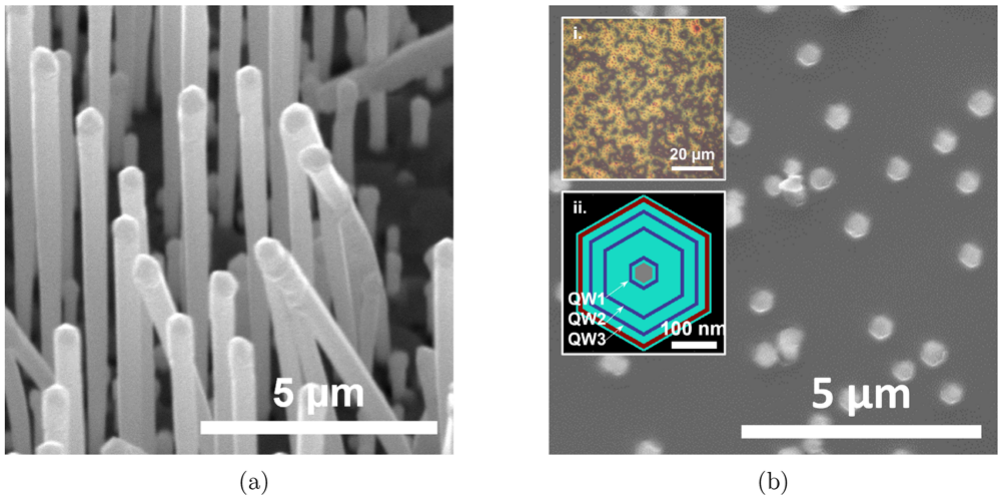
(a) SEM image of free-standing wires before nanoskiving. (b) Plane view SEM image of embedded and nanoskived nanowires. Insets: (i) Optical microscope image and (ii) 2D schematic of the NW cross-section: GaAs_0.38_P_0.62_ core (gray), GaAs QWs (dark blue, as labeled), GaAs_0.53_P_0.47_ shells (cyan), and Al_0.5_Ga_0.5_As_0.53_P_0.47_ shell (red).

Self-catalyzed GaAsP NWs (Figure 1a) were grown directly on p-type Si(111) unpatterned substrates by III–V solid-source molecular beam epitaxy (MBE).^31^ The core GaAs_0.62_P_0.38_ NW was grown with a Ga beam equivalent pressure (8.41 × 10^–8^ Torr), V/III flux ratio (∼30), P/(As + P) flux ratio (41%), substrate temperature (∼640 °C), and growth duration (1.5 h). After growth of the core, the Ga droplets were consumed by closing the Ga flux and leaving the As and P flux open. The GaAs_0.53_P_0.47_ barriers were grown with a Ga beam equivalent pressure (8.41 × 10^–8^ Torr), V/III flux ratio (50), P/(As + P) flux ratio (50%), substrate temperature (∼550 °C), and growth duration (1 h). The nominally ∼10 nm thick GaAs QW was grown between the GaAs_0.53_P_0.47_ barriers with a Ga beam equivalent pressure (8.41 × 10^–8^ Torr), V/III flux ratio (60), P/(As + P) flux ratio (50%), substrate temperature (∼550 °C), and growth duration (10 min). A further 10 nm thick Al_0.5_Ga_0.5_As_0.53_P_0.47_ shell was grown and capped with a 5 nm thin GaAs_0.53_P_0.47_ passivation layer. During growth, the substrate temperature was measured using a pyrometer.

### Nanoskiving

The NWs were cleaved by nanoskiving,^24^ a microtomy technique. Briefly, the MBE grown free-standing NWs were then embedded in low surface tension epoxy resin as a supporting matrix and cured by UV radiation (λ = 365 nm). The epoxy-supported NWs were sliced into submicron thickness films perpendicular to the NW growth direction in the  plane by the diamond knife of the ultramicrotome—an example slice is shown in Figure 1b. Sixteen cross-sectional slices were cut to thicknesses ranging between 200 and 300 nm along the NW (111) direction with further details in the Supporting Information.

### Photoluminescence Mapping

The confocal μ-PL microscopy setup used a 532 nm laser source with a 630 nm full-width half-maximum (fwhm) Gaussian spot. Samples were placed on a motor-controlled *x*–*y* stage, illuminated by a green LED from above for transmissive bright-field imaging. The laser beam passed through a Nikon MRD00205 objective lens (20× magnification, 0.75 numerical aperture) directed toward the sample–air interface. The photoluminescence passed back through the objective and was split by a pair of 50/50 beamsplitters, one taking a path to a camera for imaging and another to a 200 μm core optical fiber toward an iHR550 Horiba imaging spectrometer. The 532 nm wavelength was filtered before entering the optical fiber. The spectrometer was set to measure ±150 nm around the central wavelength of 800 nm with a 150 lines/mm grating, 0.1 mm slit, and 250 ms integration time.

The stage raster scanned over the samples with a 300 nm step size. A 100 × 100 μm^2^ PL map was taken for each sample at room temperature, taking 8 hours per scan. A dark spectrum was taken to negate from the PL spectrum to remove background signals.

### Scanning Electron Microscopy (SEM)

Scanning electron microscopy was performed using Quanta250 to acquire images for each nanoskived slice containing the GaAsP/GaAs heterostructures on a sapphire substrate. The nanowires were coated with Au/Pd [80:20] using a Quorum Q150T ES sputter coater, resulting in an average thickness of ∼6 nm to enhance conductivity in the sample. Best images were obtained using a secondary electron detector at 10.5 mm working distance and 20 keV acceleration voltage. Images acquired at 9000× magnification show ∼120 nanowires per image.

## Results and Discussion

Transmission electron microscopy (TEM) analysis has previously shown that these structures contained an 80 nm diameter GaAs_0.38_P_0.62_ core with three nominally 3.5–10 nm thick, highly compressively strained (1.7%, relative to barrier) GaAs QWs.^32^ The GaAs QWs are confined between 15 and 50 nm thick GaAs_0.53_P_0.47_ shells, and a further 15 nm thick Al_0.5_Ga_0.5_As_0.53_P_0.47_ region was grown to improve carrier confinement which was finally capped with a 5 nm GaAs_0.53_P_0.47_ passivation layer. Slices were taken from the top half of 10 μm long NWs, producing 16 planarized samples (ranging from 50 × 100 to 300 × 300 μm^2^ in area) containing 500–2000 cross-sectioned structures per slice. The nanoskiving technique allows us to investigate variations in morphology and measure the optoelectronic performance of the NWs along their length with submicron resolution with statistical confidence.

A high-resolution hyperspectral map was measured using μPL for each planarized sample, a photoluminescence (PL) spectrum per identified structure was measured and fit, and a series of parameters were extracted from the fit. Most parameter distributions were observed to be symmetric around the median value. Structures from SEM images of the slices were isolated using segmentaton and analyzed with detection algorithms, as described in the Supporting Information. Experimental results from statistical data are reported as median values with an interquartile range (IQR) to remain agnostic to the choice of distribution model. Linear fits are applied to parameters as a function of slice height, with fits tightly bound due to the use of >15,000 data points per parameter; for fit parameters, 95% confidence bands are shown.

Across all studied SEM images, around 22,000 structures were isolated using image segmentation (details in the Supporting Information). A reverse taper was observed as the diameter increased by 28_–1_^+1^ nm/μm (a relative increase of ∼5%/μm) toward the NW tips, an effect that can be observed in as-grown SEM imagery in Figure 1a. The median diameter at the lowest slice was 430_–50_^+50^ nm which increased to 590_–40_^+50^ nm at the highest slice measured, an increase of 37%. The separation between structures increased by 65_–3_^+3^ nm/μm, becoming sparser toward greater NW heights as expected: any length distribution leads to a reduction in effective density as fewer short wires are present in the planarized samples toward the tip.^25^

**Figure 2:**
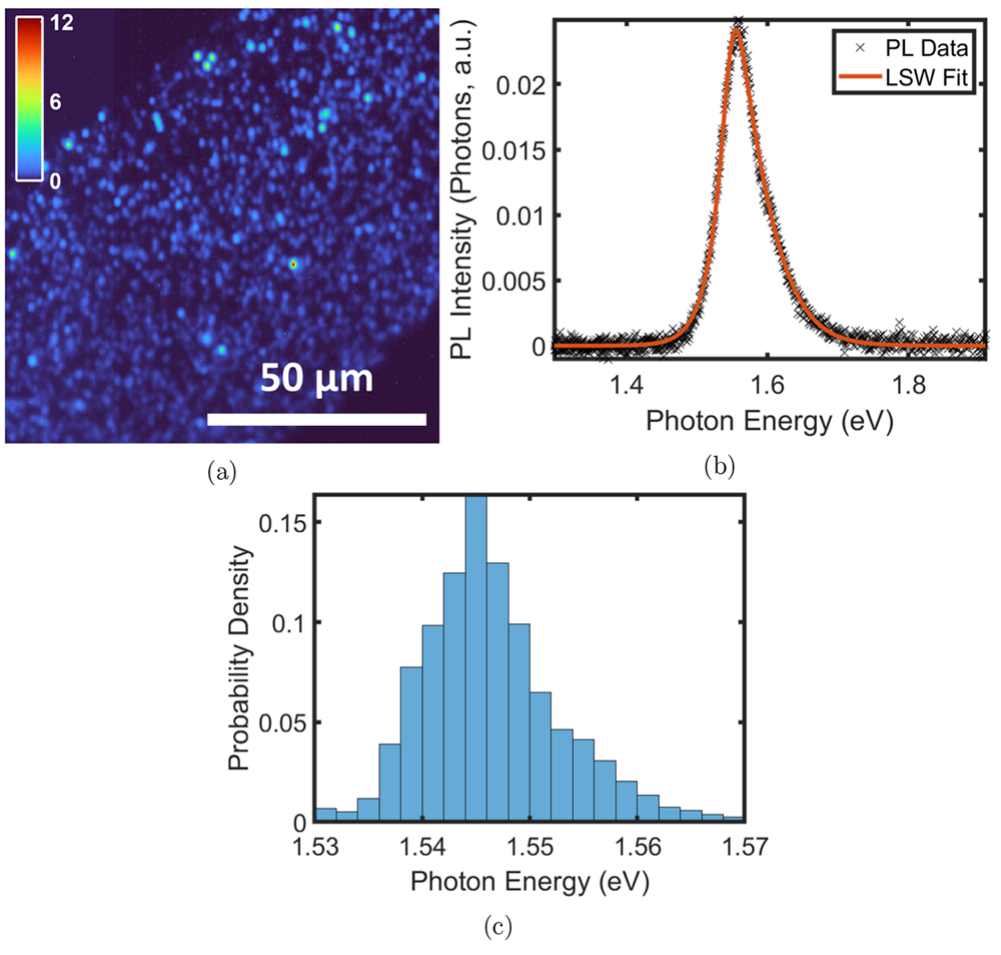
(a) 100 × 100 μm^2^ PL map [color bar representing peak PL intensity (photons, a.u.)]. (b) LSW and Urbach tail fitting of a single PL spectrum for a typical structure. (c) Transition energies for all 15,057 structures, obtained from the LSW model, showing a median at *E*_well_ = 1546_–3_^+4^ meV.

In total, 15,057 structures were identified from 2D PL intensity maps of individual slices, with an example shown in Figure 2a. Each of the 16 100 × 100 μm^2^ hyperspectral PL maps consist of 111,556 pixels, each with an associated PL spectrum, and contained ∼1000 structures as identified by image postprocessing (details of the processing, segmentation, and filtering are given in the Supporting Information). The number of detected structures from PL mapping differed from slice to slice (see the Supporting Information for details) due to variation in slice size and roughness and is not indicative of structure density; for this reason, we use SEM imagery for morphological analysis.

PL spectra for each structure were fit with a Lasher–Stern–Würfel (LSW) model as detailed in the Supporting Information.^33,34^ Briefly, the LSW model is comprised of a two-dimensional density of states model (*K*(*E*)) modified with the addition of an Urbach tail (*U*(*E*)) to account for low-energy band edge variations often observed in disordered materials,^21,35^ and convoluted with a Gaussian (*G*(*E*)) to represent the system resolution, and was given by
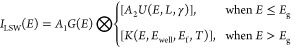
1where *E* is the photon energy, *E*_f_ is the Fermi energy, *T* is the effective emission temperature, *E*_well_ is the quantum-well transition energy, γ is the Urbach energy, and *A*_1,2_ are scaling factors. An example of a fit is given in Figure 2b.

The transition energy at the QWs (*E*_well_) was determined for each NW and was used to calculate the QW width *L* by interpolating energies from Nextnano^36^ simulations. The presence of inhomogeneous strain within the structure^18^ restricts the use of a simple infinite or finite-well approximation to calculate well widths from transition energy. A series of 2D 1-band Nextnano simulations were performed to calculate expected transition energies accounting for strain in the quantum wells. The structure was generated with a radial profile as previously described. Full details of the simulation are provided in the Supporting Information. The QWs are referred to as QW1, QW2, and QW3 from the innermost well to the outermost well (shown in Figure 1b,ii). The QW width was swept across the nominal range *L* = 3 nm to *L* = 15 nm, in 0.5 nm steps, to compare to the transition energies obtained from experiment. The simulation temperature was set at 359 K to match with the effective average emission temperature obtained from the LSW fitting, as our excitation conditions (13.6 kW/cm^2^) lead to an expected heating power of around 54 μW when considering excess photon energy (30% of incident power). While a full thermal analysis lies beyond the scope of this work, comparable experiments have determined a slightly elevated lattice temperature due to heating in the range of tens of Kelvin.^37^ Quantum eigenstates were calculated with 1-band level theory, and the energies with the highest transition probability per QW between the Γ-band and heavy-hole (HH) band wave functions were recorded.

**Figure 3:**
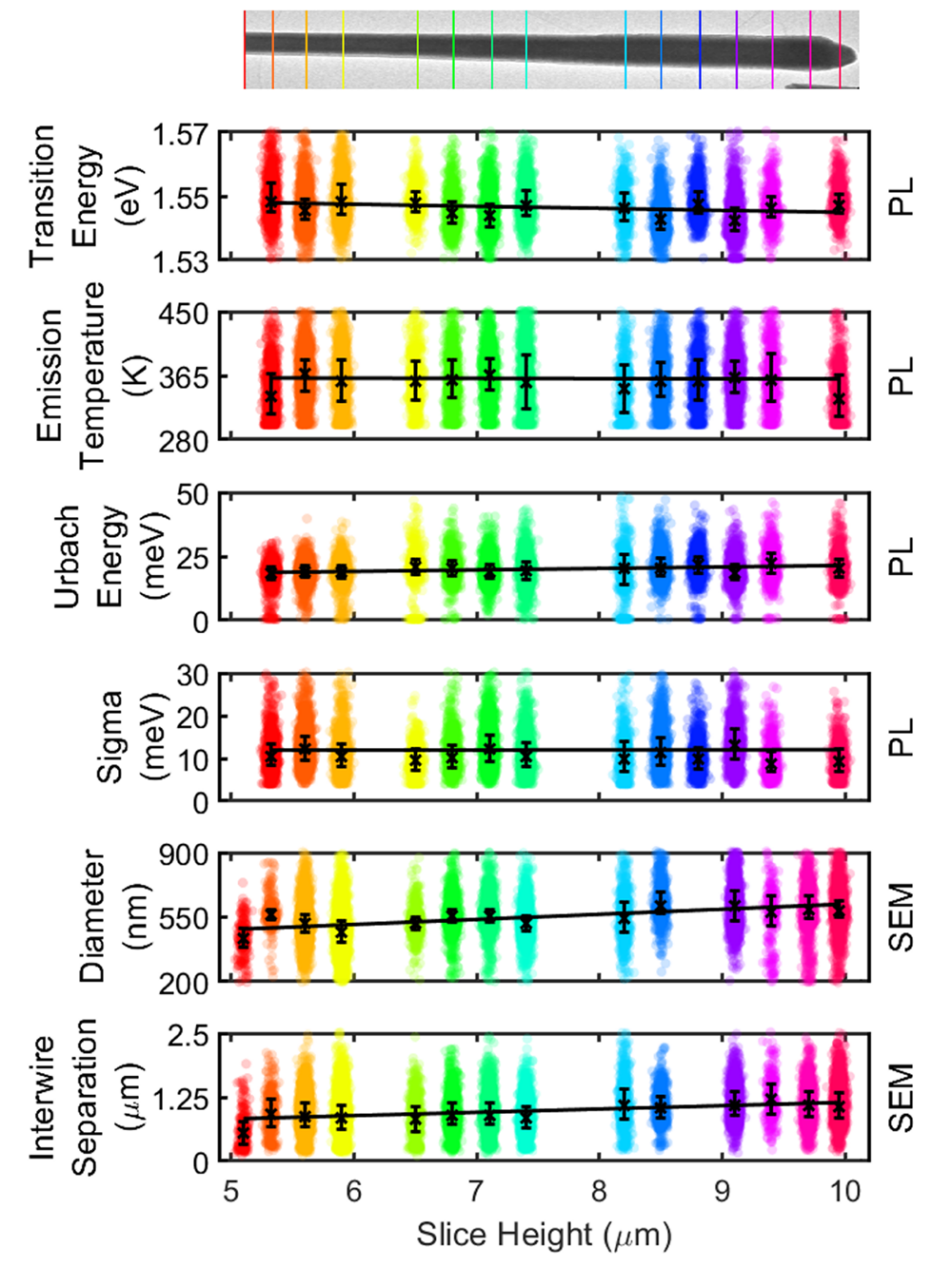
Swarm plot of properties obtained from the LSW fitting of PL spectra and SEM data. The transmission electron microscopy image (top) serves as a guide for the slice height along the NW. Black crosses are median values, with error bars representing the IQR. Best fit lines are shown as a function of slice height, and errors are referenced by 95% confidence bands in the text.

Statistics shown in Figure 2c indicate a median *E*_well_ = 1546_–3_^+4^ meV emission, associated with a median QW width of 8.2_–0.5_^+0.3^ nm across the whole population. The LSW model provides other parameters (*T*, σ, γ) as described in eq 1. These can be used to understand disorder and defect density as a function of slice height, as shown in Figure 3.

PL results show homogeneous GaAs QW emission across all slices when excited with a 2.33 eV (532 nm) source. At this excitation energy, photocarriers are generated in the wells, GaAsP barrier, and core material. The GaAsP barrier and core funnels the photogenerated carriers into the QWs, as they host a type I heterostructure.^32^ It is noted that the PL specifically probes the electronic environment at preferred recombination sites^38^ and it is possible that higher energy sites exist but are unfavorable following diffusion; when carriers reach the QW, strain efficiently confines them. The innermost QW (QW1) was found to have the highest radiative rate determined by electron–hole wave function overlap due to strain,^39^ in turn caused by a difference in lattice matching to the GaAs_0.38_P_0.62_ core containing a higher phosphorus content compared to QW2 and QW3 that are confined between GaAs_0.47_P_0.53_ shells. Simulated transition energies for QW2 and QW3 are at a slightly higher energy than QW1 by 1–10 meV. It is expected that carrier generation is weighted by volume of material that can funnel carriers into each well, as above-bandgap excitation is applied to the whole material. The majority of carriers are attributed to originate from the GaAs_0.38_P_0.62_ core which efficiently diffuse to QW1 through X-band alignment to the Γ-band edge.^32^ Nextnano simulations indicate that recombination in QW1 is most likely and supports the carrier mechanics discussed above.

While the nanoskiving process may introduce surface defects contributing to an increase in nonradiative recombination, we do not observe a difference in emission for samples of different thickness. This may be due to suppressed radiative recombination in the near surface region and a short strain relaxation length (20 nm). The ambipolar diffusion length in GaAs^40^ can reach twice the structure size of our samples; it is likely that emission will reflect inhomogeneity in emission sites rather than all sites present. Measurement of the quantum efficiency (QE) of these structures is challenging due to differing slice thickness and dielectric environment due to the supporting resin matrix; however, similar structures show a high QE of 40%.^41^

The median PL, across all measured structures, is 1546_–3_^+4^ meV and is consistent with the previous study on these structures with high Zinc Blende (ZB) purity.^18^Figure 3 shows that no large shift in transition energy is observed toward the tip of the NW; this indicates no significant increase in stacking faults,^42,43^ dislocations,^18^ and polytypism^43^ that could otherwise lower quantum efficiency.^44^ This is in agreement with reported advantages of using a liquid metal globule in self-catalyzed MBE.^43,45^

The transition energies and effective emission temperatures obtained from the LSW model (eq 1) show <1% variation along the NW. The gradient of these parameters with respect to the slice height is obtained from linear fits shown in Figure 3. The transition energy varied by −0.64_–0.08_^+0.08^ meV/μm and across the whole NW had an IQR of 7.7 meV. A slight redshift along the NW is observed, which suggests that the QW width increased toward the tip—this trend agrees with the increase in the external diameter of the structures observed in SEM but with a greatly reduced dependency.

The Urbach energy, γ = 20_–0.3_^+0.3^ meV, varied by +570_–80_^+80^ μeV/μm which relates to disorder at band edges in quantum confined materials^35^ and can be used as a proxy to characterize disorder. The increased Urbach energy along the wire indicates similarly increasing disorder toward the NW tip; this is a subtle effect which would not be observable with fewer data points, and is expected as the tip region of MBE grown NWs is typically more defective.^18,46^ Homogeneous disorder σ = 11_–3_^+3^ meV does not vary significantly over the NW length at +20_–90_^+90^ μeV/μm. This corresponds to a homogeneous spread of around 5 nm at the emission energy, which is larger than the expected spectral resolution of this system (∼1 nm). The emission line shape is dominated by the effective emission temperature, *T* = 359_–26_^+26^ K; however, this did not vary significantly (−0.3_–0.4_^+0.4^ K/μm) as a function of slice height.

**Figure 4:**
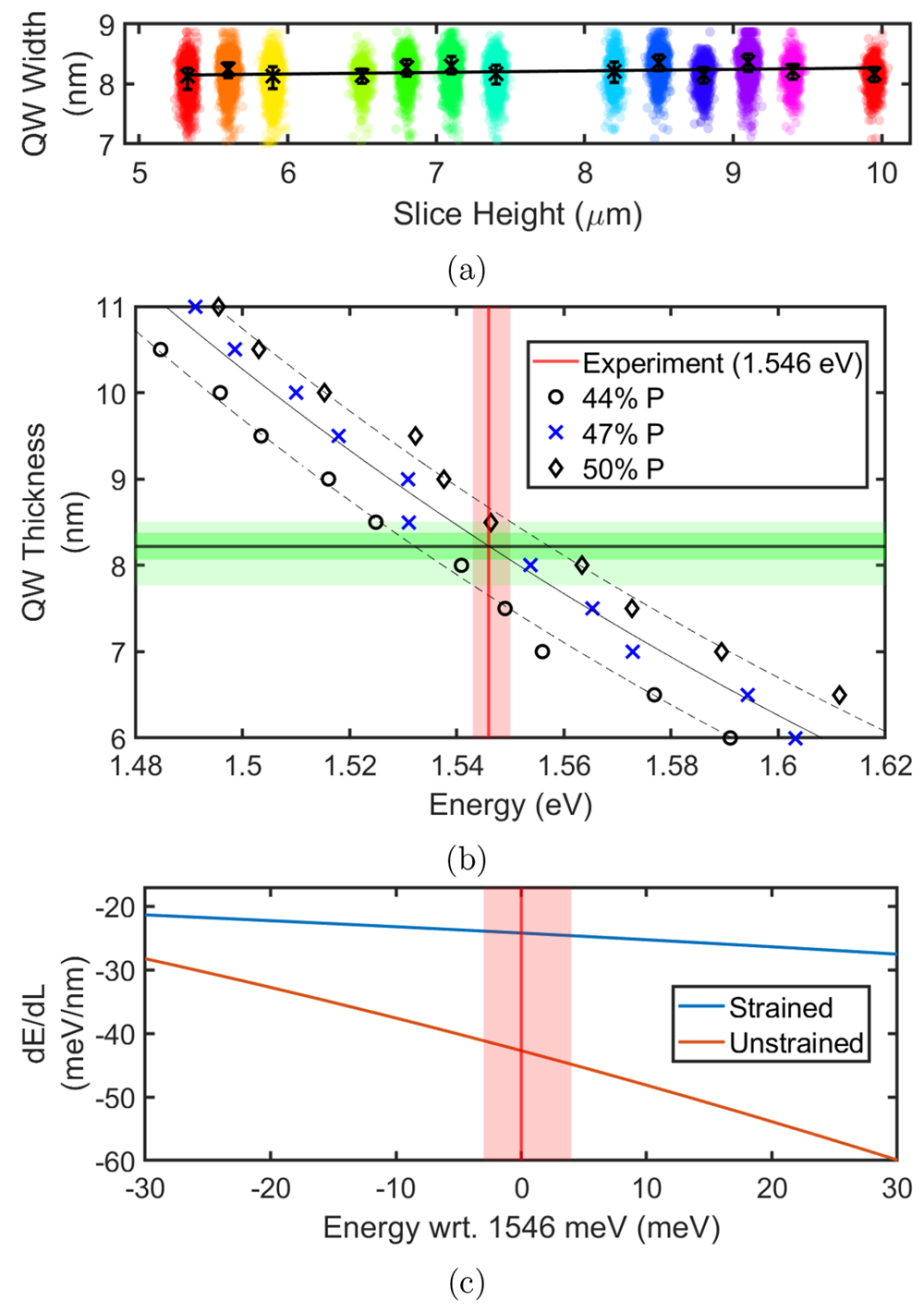
(a) Swarm plot of QW width calculated from the Nextnano simulation along the NW length. (b) Simulation sweeps of QW width and transition energy, used to extract QW widths from experimentally observed transition energies. Models for 44%, 47%, and 50% P-composition in the GaAs_0.53_P_0.47_ barriers affect the intramaterial strain and hence shift the QW energies. The dark green overlay represents the IQR of the extracted widths from our statistical analysis for *P* = 47%, whereas the light green extends to account for other P-compositions. (c) Gradient of the fit shown in (b) for strained and unstrained (data not shown) versions of the structure with GaAs_0.53_P_0.47_ barriers, showing the reduced emission dependence on QW width (and hence energy) due to strain.

The Nextnano calculations show dominant transitions occur at 1.62–1.52 eV dependent on the QW width, as shown in Figure 4b. The transition occurs between the Γ to the heavy-hole (HH) band, at the GaAs QWs. The variation of energy with QW width *L*_0_ around the observed emission energy *E*_0_, , is shown in Figure 4c and fit with a power series *L* = *AE*^*b*^ + *C*, where *A* is a scaling factor, *b* is the power term, and *C* is an offset. Through interpolation of these fits to discrete simulated energies, a QW width of *L*_0_ = 8.2_–0.5_^+0.3^ nm was obtained for *E*_0_ = 1546_–3_^+4^ meV. The QW width varied along the NW by +20_–2_^+2^ pm/μm, and while statistically significant, it is below a 0.3%/μm variation.

Two causes for the relatively small energy variation are proposed for the GaAsP/GaAs system: a reduced dependency of energy upon well width for a given emission energy due to strain effects, as indicated by the comparison between strain-aware and strain-free models in Figure 4c (i.e., a reduced ), and a high level of control over the QW thickness across the sample (a reduced Δ*L*_0_). The residual variation is likely due to the reverse taper observed in the structure, where the diameter toward the tip is greater and promotes faster radial growth.^18^ Nevertheless, most wires appear to contain a uniform QW thickness at the monolayer level. Our high-throughput methodology shows the variation for the QW width at 0.8 nm—significantly less than that of the nominal thickness target of 3.5–10 nm^18^ as seen in Figure 4 and shows the QW thickness is more tightly defined than observed from low-throughput characterization, with previous studies showing up to ±30% variation^30^ compared to ±5% in this work.

The SEM results show the overall NW morphology varies to a greater extent than the QWs. The wires exhibit a reversed taper, increasing from 430_–50_^+50^ to 590_–40_^+50^ nm, a 37% increase in median diameter which is greater than the 0.8 nm variation of the QW width, corresponding to a 10% variation. The trend in increased diameter in overall structure thickness is attributed mainly to the GaAsP barriers and passivation layers, caused by differing growth rates of the side facets^47^ which are dependent on the local III/V flux.^48^ Further, the rate of radial growth increases with the diameter of the wire, meaning the outermost shell will cause the greatest effect on morphological variation. Inverse tapering is common among self-catalyzed MBE grown wire cores and is due to gallium-rich conditions progressively increasing the diameter of the catalyst.^49^

The observed optical uniformity in the presence of overall inhomogeneous morphology and strain variations shows that appropriate design of self-catalyzed MBE grown NWs can provide a route to reduced emission disorder. However, the relative impact of inter- and intrawire inhomogeneity is dependent on the intended application.^6^ Crucially for nanowire lasing, the optimization of the diameter for the transverse mode strongly influences the lasing threshold and the interwire diameter inhomogeneity will directly impact the lasing yield.^50,51^ For the presented material, the average spread in diameter across each slice is 106 nm (19% of the nominal median); this compares to 122 nm (22%) across the whole population.

## Conclusions

In conclusion, a high-throughput methodology is presented to optically characterize 15,057 semiconductor GaAs/GaAsP core/shell heterostructures and shows that the optoelectronic uniformity achieved is superior to the morphological homogeneity.

High optoelectronic uniformity is observed in these GaAsP/GaAs QW structures, with a 1546_–3_^+4^ meV band-to-band transition associated with an 8.2_–0.5_^+0.3^ nm QW width. A 0.8 nm variation in QW thickness is observed, which represents a 10% variation across the sample size. Importantly, the emission shows a reduced dependency on variations in both QW width and overall morphology, where the median diameter increased by 37% along the NW length. This is promising for improving fabrication scalability and yield through allowing extra degrees of freedom by reducing the emission dependence on the QW width, strain, and total geometry of the structure. We therefore emphasize continued development of the radial core/shell configuration due to its promise for hosting highly strained semiconductors in applications that demand optical uniformity and high-quality material.

## Data Availability

Raw and processed spectroscopy and SEM data associated with this study is freely available via figshare at https://dx.doi.org/10.48420/22068005. Code associated with the analysis is freely available at https://github.com/p-parkinson/Improving-Quantum-Well-Tube-Homogeneity/.

## Supporting Information Available

Photoluminescence spectroscopy experimental setup, SEM image processing, photoluminescence map processing, summary table of detected structures, Lasher–Stern–Wüurfel model, and Nextnano simulation (PDF)
