# Supplementary material for: Improving Quantum Well Tube Homogeneity Using Strained Nanowire Heterostructures
Source: ACS Appl Mater Interfaces. 2023 Feb 13;15(8):10958–64. doi: 10.1021/acsami.2c22591 (PMC9982810; doi:10.1021/acsami.2c22591)
Supplement: Supplementary file 1 — am2c22591_si_001.pdf [file am2c22591_si_001.pdf]

Supporting Information: Improving Quantum Well Tube Homogeneity using Strained Nanowire Heterostructures

Nikesh Patel,[*,†] H. Aruni Fonseka,[‡] Yunyan Zhang,[¶,§] Stephen Church,[†] Ruqaiya Al-Abri,[†] Ana Sanchez,[‡] Huiyun Liu,[¶] and Patrick Parkinson[*,†]

†*Department of Physics & Astronomy, Photon Science Institute, The University of Manchester, Oxford Road, Manchester, M13 9PL, United Kingdom*

‡*Department of Physics,University of Warwick, Coventry,CV4 7AL,United Kingdom*

¶*Department of Electronic and Electrical Engineering, University College London, London, WC1E 6BT, United Kingdom*

§*School of Micro-Nano Electronics, Zhejiang University, Hangzhou, Zhejiang, 311200, China*

E-mail: nikesh.patel-3@postgrad.manchester.ac.uk; patrick.parkinson@manchester.ac.uk

Photoluminescence Spectroscopy Experimental Setup

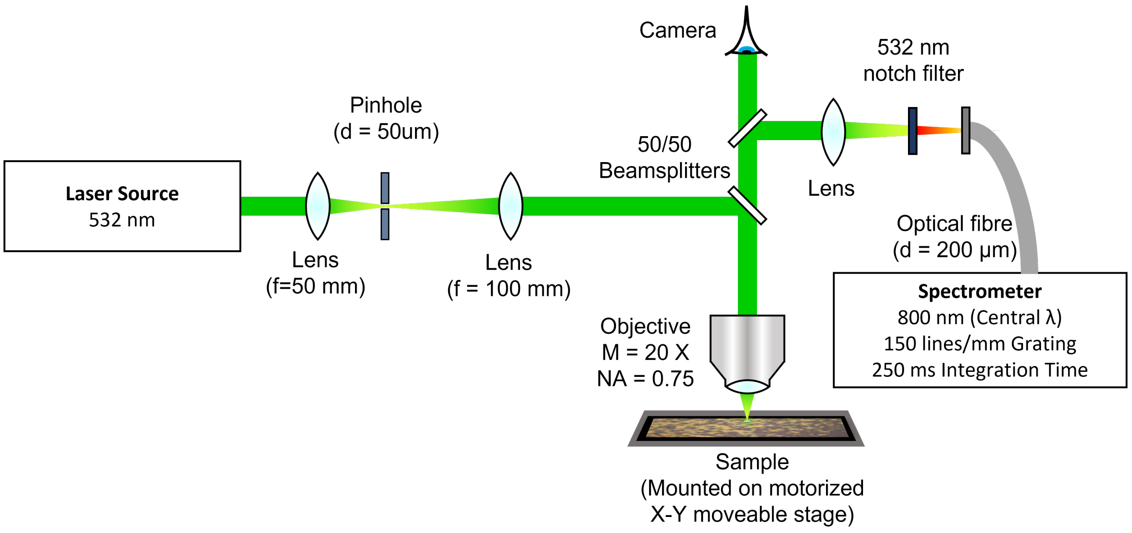

Figure S1: Confocal high-throughput photoluminescence microscopy setup.

SEM Image Processing

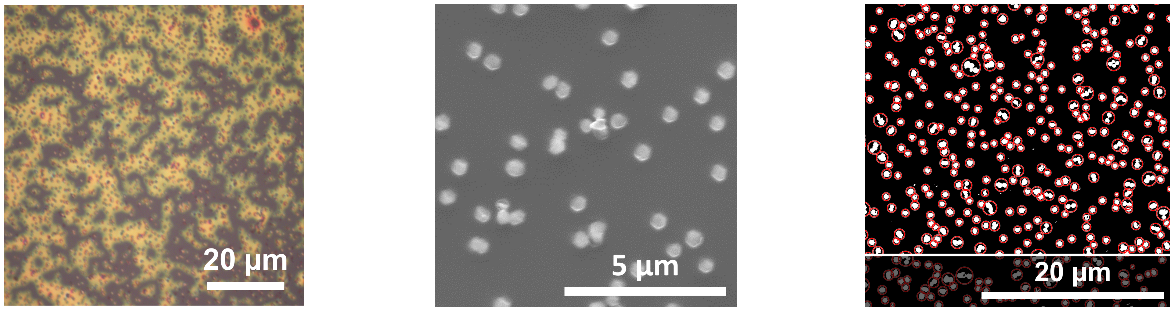

(a) (b) (c)

Figure S2: a) Optical microscope image of the heterostructure arrays, dots are structures. b) SEM magnified image of the structures, showing a clearer picture of the hexagonal facets. c) Individual identified structures in an SEM image (red circles).

The image processing toolkit from MATLAB was used to identify each structure from SEM images of the nanoskived samples (shown in Figure S2a and S2b). Specifically, we used the *regionprops* function to detect objects within a certain eccentricity range through major and minor axis and solidity.

Firstly, we binarized the images and features were detected by *regionprops*, enclosed by an elliptical region, and filtered by radii between 150 nm $< r <$ 2000 nm to remove any unphysically large or small objects, such as dust and scratches. Since our nanowires are within this lenient radius range, all isolated structures were captured by the algorithm - bar any anomalies. Grouped structures posed a problem for *regionprops* as they register as a single object, however this had a negligible effect on our dataset as they represent around 3.4% of all detected structures (objects with r > 1000 nm and <90% solidity). An example of detected objects after filtering is shown in Figure S2c. Interwire separation was calculated by picking the distance between nearest lying structures.

Photoluminescence Map Processing

Structure Segmentation via Image Processing

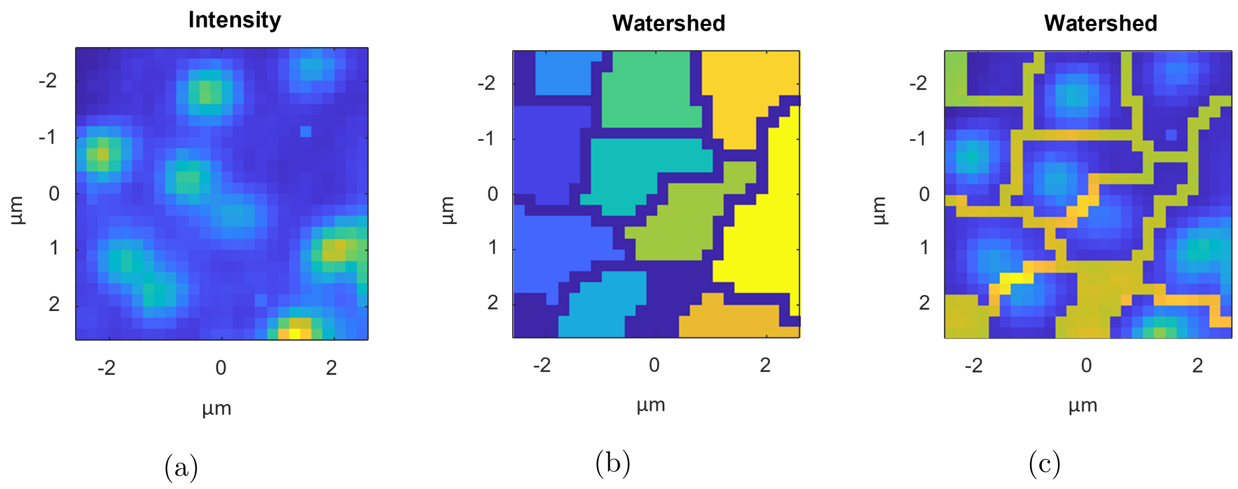

Figure S3: a) 5×5 μm 2D median intensity filtered PL map of nanoskived structures shown as regions of high intensity (yellow-green) against a blue background. b) *watershed* transform of PL map, with colours corresponding to labels. c) Inverted *watershed* mask applied to PL map to show the segmentation of individual structures.

The 100×100 μm 2D PL maps were processed to isolate individual structures based on integrated PL intensity by using MATLAB's *watershed* transform. The 2D PL maps (e.g.

Figure S3a) represent a hyperspectral image of emission intensity, where each pixel is associated with an individual PL spectrum. A 2D median filter was applied in the spatial domain to remove anomalously intense peaks. The *watershed* transform segments images based on their intensity, treating the image as a surface with catchment basins separated by a *watershed* line which lies at a computed threshold of the background intensity, to create a mask (Figure S3b) to isolate individual structures when applied to the hyperspectral PL map (Figure S3c). Regions containing more than 100, or less than 20 pixels, were filtered out as they suggest unphysical structure dimensions outside of the nominal 300-600 nm range. PL spectra per pixel in each masked region was averaged so one PL spectra represented a single structure. A total of 21,480 PL spectra was fitted with the LSW photoluminescence model. 6,174 unphysical fits (28.7%) were removed from the dataset based on PL parameters as follows: $1.50\,\mathrm{eV} < E_{Well} < 1.60\,\mathrm{eV}$, $270\,\mathrm{K} < T < 500\,\mathrm{K}$, $0\,\mathrm{eV} < \sigma < 0.5\,\mathrm{eV}$.

Summary Table of Detected Structures

Table S1: Detected structures per slice in ($100 \times 100\,\mu m$) PL maps and SEM images. Value with N/A: This sample was not measured.

Height along NW [μm]	Nominal Slice Thickness [nm]	No. Structures (PL Map)	Density [μm^{-2}] (SEM)
5.10	200	82	1.8374
5.33	250	1024	1.0928
5.60	300	1899	1.616
5.90	300	1436	1.1814
6.20	300	N/A	N/A
6.50	300	1070	1.2026
6.80	300	1876	1.1030
7.10	300	1393	1.1038
7.40	300	497	1.1970
8.20	300	1328	0.9159
8.50	300	832	0.9699
8.80	300	1626	N/A
9.10	300	647	0.9222
9.40	300	167	0.8296
9.70	300	167	0.9254
9.95	200	781	0.9322

PL data for slices at 5.10 μm and 9.70 μm were excluded due to slice contamination where >80 % of data points were rejected.

Lasher-Stern-Würfel Model

The Lasher-Stern-Würfel[1,2] (LSW) model predicts the band-to-band transition of a photoluminescence peak from radiative carrier recombination. The PL spectra we measure consists of a single peak, therefore we attribute this to a band-to-band transition, and is appropriately fit by the LSW model. In this section, LSW model is explained where we apply two modifications:

1. An Urbach tail is added to account for band-edge variations, affecting the fit at lower energy parts of the PL spectrum ($E < E_{Well}$), where E_{Well} is the PL transition energy, originating from sub-bandgap states in disordered materials.[3]

2. A Gaussian is convoluted with the model to account for energy variations introduced by the laser source and detection system.

The unmodifed LSW model (equation 1), is based on the Planck-Einstein radiation law.[4]

$$I_{PL}(E) = \frac{2\pi}{h^3c^2}\frac{E^2a(E)}{\exp\left(\frac{E-\Delta\mu}{k_BT}\right)-1}, \tag{1}$$

where $a(E)$ is the absorptivity, $\Delta\mu$ is the quasi-Fermi level splitting of electrons and holes (a term to express the external flux of spontaneous emission, under generalised non-equilibrium conditions) and T is the electron temperature. The absorptivity is given as $a(E) = 1 - \exp(\alpha(E)d)$, dependent on the absorption coefficient, $\alpha(E)$ under a parabolic band assumption, and a characteristic length, d, over which carriers are generated and radiatively recombine.[5]

An implication of the parabolic band assumption is that the absorption coefficient scales proportionally to the Joint Density of States (JDOS) that is itself proportional to the square root of energy (JDOS $\propto \sqrt{E - E_{Well}}$), which leaves $\alpha_0(E) = \mathrm{C}\sqrt{E - E_{Well}}$,[6] assuming the transition matrix element is between initial and final states,[5] where E_{Well} is the transition energy and C is a fitting parameter which absorbs parameter d.

The absorption coefficient strongly depends on the level of excitation which requires a correction factor to prevent the excited states contribution to absorption.[7] This is given by the difference between the Fermi-Dirac distribution of holes and electrons, $(f_v(E, \mu_h) - f_c(E, \mu_e))$, which can be expressed in terms of the quasi-Fermi level splitting, $\Delta\mu$. The Boltzmann approximation is used for this expression in equation 2.

$$f(E, \Delta\mu, T) = f_v - f_c = 1 - \frac{2}{\exp\left(\frac{E-\Delta\mu}{2k_BT}\right)+1} \tag{2}$$

The Urbach tail, $U(E)$ shown in equation 3, is taken in to account by convolution with

α_0.

$$U(E, E_{Well}, \gamma) = N \exp\left(-\frac{E_{Well} - E}{\gamma}\right), \tag{3}$$

where N is a normalization constant, E_{Well} is the transition energy and γ is the Urbach energy which determines the width of the tail.

Putting this altogether, we have a corrected expression for the absorptivity (equation 4).

$$a(E, E_{Well}, \Delta\mu, T, \gamma, C) = 1 - \exp\left[-\left[\alpha_0(E, E_{Well}, C) \circledast U(E, E_{Well}, \gamma)\right] f(E, \Delta\mu, T)\right] \tag{4}$$

The final LSW model is convoluted with a Gaussian, $G(E) = \exp\left(-\frac{E - E_{Well}}{\sigma^2}\right)$ giving the finalised equation 5:

$$I_{PL}(E) = \frac{2\pi}{h^3 c^2} \frac{E^2 a(E) \circledast G(E)}{\exp\left(\frac{E - \Delta\mu}{k_B T}\right) - 1}$$

$$I_{PL}(E) = \frac{2\pi}{h^3 c^2} \frac{E^2}{\exp\left(\frac{E - \Delta\mu}{k_B T}\right) - 1} \left[1 - \exp\left(-\left[\alpha_0(E) \circledast U(E)\right] f(E)\right)\right] \circledast G(E)$$

$$\begin{aligned} I_{PL}(E) = & \frac{2\pi}{h^3 c^2} \frac{E^2}{\exp\left(\frac{E - \Delta\mu}{k_B T}\right) - 1} \times \\ & \left(1 - \exp\left[-NC \int_{-\infty}^{+\infty} du \ \exp\left(-\frac{E_{Well} - E}{\gamma}\right) \sqrt{(E - E_{Well}) - u}\right]\right) \times \\ & \left(1 - \frac{2}{\exp\left(\frac{E - \Delta\mu}{2 k_B T}\right) + 1}\right) \circledast \exp\left(-\frac{E - E_{Well}}{\sigma^2}\right), \end{aligned} \tag{5}$$

Where $u = E' - E_{Well}$.

Nextnano Simulation

A 2D nextnano simulation from the GaAsP core to the outermost GaAsP barrier was set with x, y and z simulation planes defined in the $(01\bar{1})$, $(\bar{2}11)$ and $(\bar{1}\bar{1}\bar{1})$ directions, respectively. The x and y planes correspond to the side facets and the z plane to the growth direction. All crystals were simulated under minimized strain (relative to the $GaAs_{0.53}P_{0.47}$ barriers) in the pure ZincBlende (ZB) phase with a uniform 1 nm grid spacing and temperature at 359 K to match the observed emission temperature.

Transition energies were obtained from quantum mechanical calculations based on 1-band theory. Transitions were extracted based on the most probable transitions in QW1 (the inner QW), due to its favorable lower bandgap due to strain compared to QW2 and QW3. These energies were obtained from the difference in energy of the corresponding quantum wavefunctions in the Γ-band and heavy-hole band with the greatest transition probability.

Strain Relaxation

Nextnano simulations[8] were run in 2D to examine the nature of strain relaxation as shown in Figure S4. Nanoskiving cleaves across the $[\bar{1}\bar{1}\bar{1}]$ direction, exposing the GaAs QWs and allows strain relaxation due to an absence of material to maintain the lattice mismatch. Figure S5a shows the increasing relaxation at the QW/air interface as QW thickness increases.

Since strain is an important effect for carrier confinement[9,10] and, to some degree, affects the local band structure of the material[11] it is possible that optoelectronic properties are modified in the local structure. Further, since our growth is in the $[\bar{1}\bar{1}\bar{1}]$ direction - piezoelectric effects may need to be considered[12] which cause redshifting.[10]

Figure S6 shows the variation of the classically calculated Gamma-HH bandgap within the structure - this is below transition energy measured in PL as it does not consider carrier confinement. Carriers localise to mid-facet well regions rather than hexagonal corners due to the lower bandgap energy.

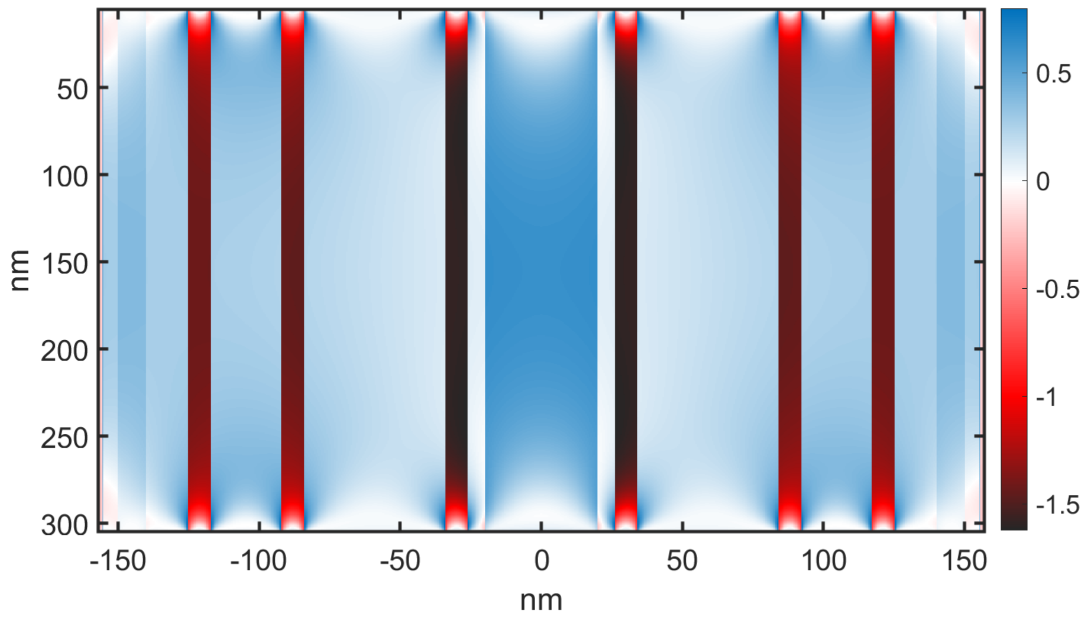

Figure S4: e_{ZZ} strain in the XZ-plane (along $[\bar{1}\bar{1}\bar{1}]$ growth direction) of the GaAs/GaAsP heterostructure with 8 nm QW thickness. Nanoskived interfaces are at $y = 5$ nm and $y = 305$ nmm, where the strain relaxes from 1.7% to 0% compressive strain. Colorbar represents strain (%).

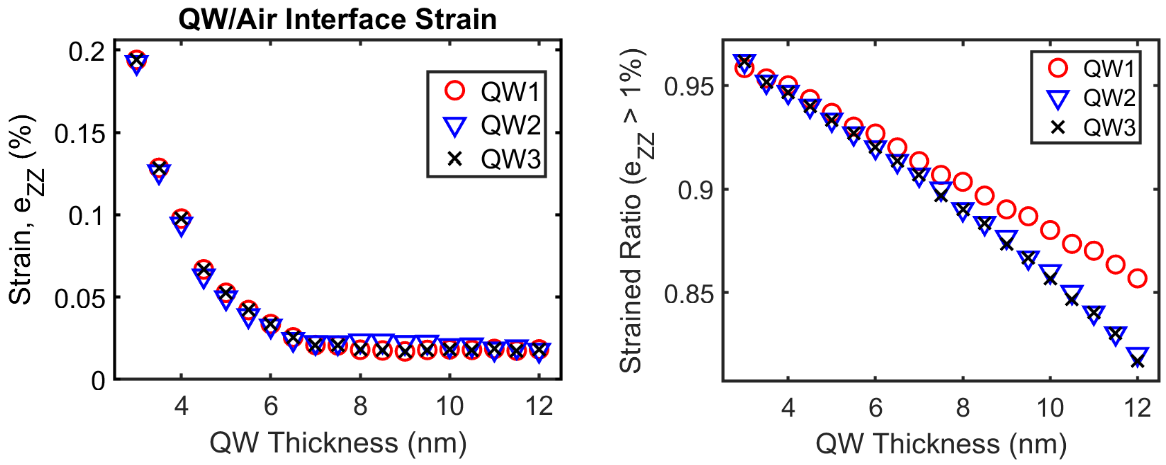

(a) (b)

Figure S5: a) Compressive strain of GaAs QWs at the nanoskived cleaving plane. b) Proportion of highly strained (> 1%) regions sampled in the QWs occuring due to strain relaxation at the cleaved surfaces.

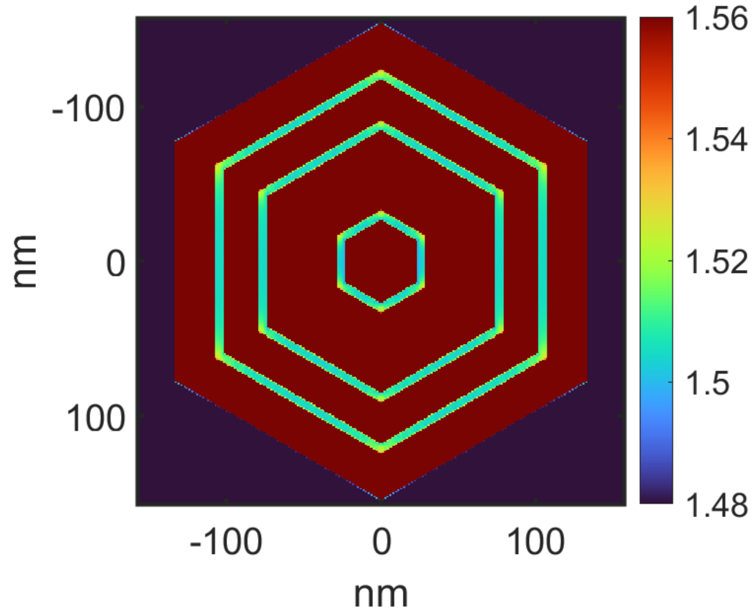

Figure S6: Gamma-HH bandgap in a planar cross-section simulation. The lowest bandgap within the QWs appears in edges (~1.50 eV) rather than corners (~1.52 eV), facilitating carrier localization in the edges. The scale bar represents bandgap energy (eV).

References

(1) Lasher, G.; Stern, F. Spontaneous and Stimulated Recombination Radiation in Semiconductors. *Physical Review* **1964**, *133*, A553–A563.

(2) Wurfel, P. The Chemical Potential of Radiation. *Journal of Physics C: Solid State Physics* **1982**, *15*, 3967–3985.

(3) Urbach, F. The Long-Wavelength Edge of Photographic Sensitivity and of the Electronic Absorption of Solids. *Physical Review* **1953**, *92*, 1324–1324.

(4) Einstein, A. The Quantum Theory of Radiation. *Physikalische Zeitschrift* **1917**, *18*, 121 – 128.

(5) Katahara, J. K.; Hillhouse, H. W. Quasi-Fermi Level Splitting and Sub-bandgap Absorptivity from Semiconductor Photoluminescence. *J. Appl. Phys* **2014**, *116*, 173504.

(6) Fadaly, E. M. et al. Direct-bandgap emission from hexagonal Ge and SiGe alloys. *Nature* **2020**, *580*, 205–209.

(7) Bhattacharya, R.; Pal, B.; Bansal, B. On Conversion of Luminescence into Absorption and the van Roosbroeck-Shockley Relation. *Applied Physics Letters* **2012**, *100*.

(8) Birner, S.; Zibold, T.; Andlauer, T.; Kubis, T.; Sabathil, M.; Trellakis, A.; Vogl, P. Nextnano: General purpose 3-D simulations. *IEEE Transactions on Electron Devices* **2007**, *54*, 2137–2142.

(9) Zhang, Y.; Saxena, D.; Aagesen, M.; Liu, H. Toward Electrically Driven Semiconductor Nanowire Lasers. *Nanotechnology* **2019**, *30*, 192002.

(10) Hocevar, M.; Thanh Giang, L. T.; Songmuang, R.; Den Hertog, M.; Besombes, L.; Bleuse, J.; Niquet, Y. M.; Pelekanos, N. T. Residual Strain and Piezoelectric Effects in Passivated GaAs/AlGaAs Core-Shell Nanowires. *Applied Physics Letters* **2013**, *102*, 191103.

(11) Bahder, T. B. Eight-band k.p Model of Strained Zinc-blende Crystals. *Physical Review B* **1990**, *41*, 11992–12001.

(12) Vurgaftman, I.; Meyer, J. R.; Ram-Mohan, L. R. Band Parameters for III–V Compound Semiconductors and their Alloys. *Journal of Applied Physics* **2001**, *89*, 5815–5875.
